# Our Lady Competitors

**Published:** 1901-08-31

**Authors:** 


### The Hospital

A Journal of

The Medical Sciences and Hospital Administration.

Vol. XXX.—No. 779.] Edited by Sir Henry Burdett, K.C.B., and Solomon C. Smith, M.D., M.R.C.P. [August 31, 1901.

### Our Lady Competitors.

When ladies first began to seek entry into the medical profession there were plenty who prophesied evil both in regard to the ladies and their new vocation. No sooner, however, had their admission become an accomplished fact than much of the outcry died away. That became apparent which ought to have been seen before, namely, that medical women were not such dreadful creatures after all, and that the knowledge gained by a medical education no more detracts from the modesty of woman than it does from the chivalry of man. It also quickly became evident, at any rate in regard to the pioneers, that the "intruders" were mostly women of exceptional powers, and that their object was not always mere bread and butter, but rather the advancement of what they supposed to be the cause of their sex. Moreover, for a long time they were few in number, and what with educating their fellows and attending to those delicate people who regarded the detailing of their symptoms to male doctors as a disagreeable and even indecent proceeding, there was plenty for them to do, and thus the question of competition hardly arose.

As years have rolled by, however, all the glamour of oddity has departed, and the lady doctor, like her brethren, has become a very ordinary person who, also like many of her brethren, regards doctoring as a mere means of earning a living. Nor have the public been backward in doing the same. In fact they have "gone one better," and applying to these enterprising ladies the same criterion as that by which female labour is almost universally appraised, have come to look upon them as cheap doctors. It is a great pity. We are infinitely grieved to think that after undergoing the not altogether charming life and experiences of the medical student, ladies should find themselves in such straits as to be ready to snatch at any chance of keeping a roof over their heads, and as mere males, perhaps, we are not entirely free from an uncomfortable feeling that, as in all other kinds of work, the outcome of all this must be a still further diminution of the already small remuneration obtained by those engaged in general practice. But we cannot shut our eyes to the tendency which, within the last year or two, has made itself manifest for people to seek the help of medical women under circumstances which appear to indicate that price is at the bottom of the predilection.

And it must be so. The thing was foreseen, and though for a time it was forgotten, the downgrade competition is sure to come. A man is driven by male instincts. He not only seeks a roof and a dinner but the power of supporting a wife and family. A woman is different. It may not have been so with the pioneers who, as we have said, were exceptional in many ways, but by this time the medical schools are recruited from among average women, and average woman looks, not to support a family, but to keep herself in as good a position as may be till the husband comes along who will do this disagreeable duty for her; and if perchance the husband does not come along, then she is glad to accept what will support her single self in reasonable comfort. Thus in every trade, business, and profession the superior remuneration of man is fixed partly (forgive us, dear ladies, for so saying) by his superior efficiency and partly by the inferior demands of woman. On every side we find this sort of competition going on; between married men with large responsibilities on the one side, and single women with practically no responsibilities, or only such as are limited to their own personal wants, on the other. Hitherto medical men have not to any appreciable extent felt the pressure arising from this cause, nor are they likely to do so for a time. The numbers are too small. But it will come, and to a large extent the form which it will take will probably depend upon the relations which are encouraged to grow up between medical men and medical women.

Among medical men there are undoubtedly many sets and many grades, just as there are many divisions in accordance with the class of work undertaken, and so it must be with medical women. But we cannot help seeing that, in addition to all this, a line of cleavage in relation to sex is tending to show itself which is likely in the end to act injuriously upon the profession. It is an infinite pity that medical women are not allowed to become members of our various medical societies. No harm has happened to the British Medical Association from the admission of women, and no harm ought to happen from their admission to the other more purely scientific medical societies. At present there is very little to force upon medical women any idea of the solidarity of the profession—in fact some of them must find it difficult to see in what sense they are members of a professional body at all—and there is much to tempt them to play each for her own hand. This is to a large extent our own fault. We do nothing rude to these ladies, we are quite polite to them when they are thrown in our way, we accept them with resignation as one among the various pin pricks devised by a kind providence, and we gently ostracise them.

In so doing we shall have our reward. The ladies already occupy such a position as to make it almost an indignity that they should have to ask to be admitted to our societies; shortly they will establish a group of special societies of their own—a beginning has already been made—and instead of the medical profession being one we shall see it divided by a great cleavage, not on the basis of science, but of sex.

